# No significant viral transcription detected in whole breast cancer transcriptomes

**DOI:** 10.1186/s12885-015-1176-2

**Published:** 2015-03-18

**Authors:** Danai Fimereli, David Gacquer, Debora Fumagalli, Roberto Salgado, Françoise Rothé, Denis Larsimont, Christos Sotiriou, Vincent Detours

**Affiliations:** 1 IRIBHM – Université Libre de Bruxelles, ULB, Campus Erasme CP602, 808 route de Lennik, 1070 Brussels, Belgium; 2 WELBIO, 808 route de Lennik, 1070 Brussels, Belgium; 3 Breast Cancer Translational Research Laboratory, Institut Jules Bordet, Université Libre de Bruxelles (ULB), Bld de Waterloo, 125-1000 Brussels, Belgium; 4 Department of Pathology, Institut Jules Bordet, Université Libre de Bruxelles (ULB), Bld de Waterloo, 125-1000 Brussels, Belgium

**Keywords:** Breast cancer, Virus discovery, Next-generation sequencing, RNA-seq, Exome

## Abstract

**Background:**

Studies evaluating the presence of viral sequences in breast cancer (BC), including various strains of human papillomavirus and human herpes virus, have yielded conflicting results. Most were based on RT-PCR and *in situ* hybridization.

**Methods:**

In this report we searched for expressed viral sequences in 58 BC transcriptomes using five distinct *in silico* methods. In addition, we complemented our RNA sequencing results with exome sequencing, PCR and immunohistochemistry (IHC) analyses. A control sample was used to test our *in silico* methods.

**Results:**

All of the computational methods correctly detected viral sequences in the control sample. We identified a small number of viral sequences belonging to human herpesvirus 4 and 6 and Merkel cell polyomavirus. The extremely low expression levels—two orders of magnitude lower than in a typical hepatitis B virus infection in hepatocellular carcinoma—did not suggest active infections. The presence of viral elements was confirmed in sample-matched exome sequences, but could not be confirmed by PCR or IHC.

**Conclusions:**

Our results show that no viral sequences are expressed in significant amounts in the BC investigated. The presence of non-transcribed viral DNA cannot be excluded.

## Background

Various risk factors have been linked with breast cancer, including sex, age, family history of cancer, radiation and others [1]. However, the underlying mechanisms in breast carcinogenesis are not fully understood. Viral infections have long been considered a risk factor in several types of cancer. For example, the human papillomavirus (HPV) contributes to cervical and head and neck cancer, while the human herpesvirus 4 (EBV) contributes to Burkitt’s lymphoma. Potential carcinogenetic mechanisms include expression of viral oncogenes or inactivation of tumor suppressors. For example, in cervical cancer the expression of HPV viral oncoproteins E6 induces the degradation of the tumor suppressor gene p53 [2].

Numerous investigators have tried to establish a link between breast cancer and viral infections. The results, however, remain conflicting. Di Lonardo et al. [3] detected HPV DNA in nearly 30% of ductal breast carcinomas when using polymerase chain reaction (PCR). A number of studies from various research groups followed. Viruses that have been mainly detected and linked to breast cancer include HPV types 16 and 18, EBV and human herpesvirus type 5 (CMV) [4-9]. In contrast, studies were published that failed to detect viral sequences in breast cancer [10,11]. Apart from the above viruses, the mouse mammary tumor virus (MMTV) has also been in the center of attention, due to its link with mammary cancer in mice, with a recent study detecting MMTV sequences in the milk of women who had undergone breast biopsies [12]. The review of Salmons et al. [13] points out, as mentioned above for the other viruses, the controversy in the results between studies for the presence or not of MMTV in breast cancer and the fact that viral sequences are difficult to detect in tumors.

The majority of the above studies relied on PCR or *in situ* hybridization (ISH). These technologies require prior assumptions about which viruses might be associated with breast cancer. In contrast, next generation sequencing (NGS) technologies make it feasible to directly detect viral sequences without any *a priori* assumption regarding the virus involved. A small number of studies have exploited transcriptome NGS in order to detect viral sequences in cutaneous squamous cell carcinoma, non-Hodgkin’s Diffuse Large B-Cell Lymphoma and other cancer types [14,15]. Recently, two studies have scanned transcriptome sequences from The Cancer Genome Atlas (TCGA), which contains several thousands of human samples from 20 cancer types, for transcribed viral sequences [16,17]. No viral sequence could be detected in the pool of TCGA breast cancers examined in the two studies, 750 and 810, respectively.

There are currently a number of computational algorithms available for detecting pathogen sequences using NGS data for example PathSeq [18] or VirusSeq [19]. The majority of them involve an initial step of subtraction of human sequences and the subsequent alignment of the remaining non-human sequences to a database of pathogen sequences (which can include viral, bacterial or fungal sequences). The differences between these tools are based mainly on the aligners used at each step of the procedure, which can produce varying results.

The aim of this study was to investigate the presence of viral transcripts in a cohort of breast cancer samples encompassing the known main molecular subtypes (luminal A and B, triple negative and HER2 positive). In order to accomplish our goal, we performed RNA sequencing and implemented five different but complementary *in silico* methods covering a range of available bioinformatics techniques. In addition, we matched NGS results against PCR and immunohistochemistry (IHC).

## Methods

### Samples selection

A total of 58 breast cancer (BC) patients for whom fresh-frozen tumor and normal, adjacent material as well as formalin-fixed, paraffin embedded (FFPE) tumor material was available at Bordet Tumor Bank (Jules Bordet Institute, Brussels, Belgium) were selected for this project. Patients were recruited between 2007 and 2011 and associated clinico-pathological data are available for all.

The use of the data is consistent with the informed consent signed by the patients or has been granted ethical approval by the local Ethics Committee and is in accordance with the applicable laws and regulations of Belgium. The study was approved by the ethics committee of Institut Jules Bordet (study number: CE1967).

### Samples histopathology

On the basis of their immunohistochemistry (IHC) profile, patients were classified in one of the four main IHC BC subtypes: triple negative (TN: estrogen receptor (ER), progesterone receptor (PgR), and human epidermal growth factor receptor 2 (HER2) negative), HER2 positive (any ER and PgR, HER2 positive), luminal A (ER positive, HER2 negative, histological grade 1) and luminal B (ER positive, HER2 negative, histological grade 3).

### RNA extraction

RNA from fresh-frozen material was extracted using TRIzol® (Life Technologies, Carlsbad, California) following the manufacturer’s instructions. RNA concentration was defined using the NanoDrop 1000 (Thermo Scientific, Waltham, Massachusetts), and RNA integrity (RIN: RNA Integrity Number) was assessed using an Agilent 2100 Bioanalyzer (Agilent Technologies, Santa Clara, California). All the samples yielded enough material for downstream analyses and had a RIN equal or superior to 6.5.

### DNA extraction

DNA from both tumor and normal fresh-frozen material was extracted using DNeasy Blood and Tissue kit® (Qiagen, Venlo, Netherlands) following the manufacturer’s instructions. DNA concentration was measured using the NanoDrop 1000 instrument (Thermo Scientific). All the samples yielded enough material for downstream analyses.

### RNA sequencing

Transcriptome sequencing was performed at DNAVision (Gosselie, Belgium). Transcriptome libraries were constructed using the Illumina® TruSeq™ RNA Sample Preparation Kit for paired end reads sequencing on the HiSeq 2000 (Illumina, San Diego, California) following the manufacturer’s instructions.

Briefly, starting from 1 μg of total RNA, the poly-A containing mRNA molecules was purified using poly-T oligo-attached magnetic beads. Following purification, the mRNA was fragmented into small pieces using divalent cations at elevated temperature. The cleaved RNA fragments were copied into first strand cDNA using reverse transcriptase and random primers. This was followed by second strand cDNA synthesis using DNA Polymerase I and RNase H and purification using the AMPure XP beads (Agencourt BioSciences Corporation, Beverly, Massachusetts). The cDNA fragments went through an end repair process, the addition of a single ‘A’ base and ligation of the adapters. The products were purified using the AMPure XP beads and enriched with PCR (15 cycles) to create the final cDNA library followed by purification using the AMPure XP beads. Libraries’ quality control and quantification were performed using the Agilent Bioanalyser 2100 and qRT-PCR; libraries were pooled (4 libraries/pool). Clusters were generated in a cBot Cluster Generation System using the Paired-End Cluster Generation Kit v2-HS and sequenced on the Illumina HiSeq 2000 platform (Illumina) with a 2x50 base-pairs (BP) paired-end mode.

### Exome sequencing

Exome sequencing was performed at GATC (Konstanz, Germany). Genomic libraries from the tumor and matched normal samples were generated using the Illumina Paired End DNA sample preparation kit (Illumina) following the manufacturer’s instructions. Enrichment was performed using the Agilent SureSelect Human All Exon V3 kit (Agilent) following the manufacturer’s instructions.

Briefly, 2–3 μg of total genomic DNA was randomly fragmented to between 150 and 600 bp by focused acoustic shearing (Covaris Inc, Wouburn, Massachusetts). A cleanup was performed using AMPure beads (Agencourt BioSciences Corporation) following the manufacturer’s protocol and the material quality was assessed using the Agilent Bioanalyser 2100 (Agilent).

The size-fractionated DNA was end repaired using T4 DNA polymerase, Klenow polymerase and T4 polynucleotide kinase and purified using AMPure beads. The resulting blunt ended fragments were A-tailed using a 3′-5′ exonuclease-deficient Klenow fragment, purified using AMPure beads and ligated to Illumina paired-end adaptor oligonucleotides in a ‘TA’ ligation at 20°C for 15 minutes. The product was purified using AMPure `beads. After estimation of the concentration, the adaptor-ligated library was amplified and then purified using AMPure beads. Quality and quantity were assessed using a 2100 Bioanalyzer (Agilent).

The enriched regions were captured, purified, PCR amplified and purified using AMPure beads. After quantification and quality control of the captured library, samples were pooled (four samples/lane) for loading on an Illumina HiSeq 2000. Samples were sequenced in paired-end mode, with a read length of 2x100 bases.

### Transcriptome and exome read mapping

RNA-seq reads were mapped with the Burrows-Wheeler Aligner [20] (BWA v0.5.9) simultaneously on the human reference genome (hg19) and a library of splice junctions. Reads were mapped with command ‘bwa aln –n 6’ to report up to 6 matches per reads with multiple matches so that read pairing could be performed with a custom perl script considering the true distance between mates after removal of intronic regions between them. Further, we removed all non-unique and discordant read pairs. The splice junctions library was constructed by concatenating respectively the last and first 50 nucleotides for each pair of consecutive exons. We used gene annotations from Refseq, UCSC, Ensembl and Gencode, downloaded from the UCSC Table Browse [21]. Exome-seq reads were also mapped to the hg19 reference genome using BWA, with default options. For both transcriptome and exome alignments, we further removed duplicates with Picard’s MarkDuplicates utility (http://broadinstitute.github.io/picard) (v1.59) and performed local realignment using the GATK’s IndelRealigner program [22] (v1.4-15).

### Computational detection of viral sequences using RNA-Seq

In pipeline 1, all reads not mapped to the human genome or human splice junctions were aligned to the RefSeq database of viral genomes (n = 4537), with BWA (v0.6.1) with default parameters. All reads that aligned to a viral genome were considered as potential viral reads and were further aligned using blastn [23] (v2.2.28) (with default parameters) against the NCBI nucleotide (nt) database. Reads with the best blast hit (lowest e-value and highest alignment score) matching the BWA virus hit were considered of true viral origin.

In pipeline 2, unmapped reads were aligned to the RefSeq viral database with the use of blastn instead of BWA (blastn was run with default values and e-value of e-05). All reads that aligned to a viral genome were considered as potential viral reads and were further aligned to the nt database as in the previous pipeline.

In pipeline 3, we performed a *de novo* assembly on the unmapped reads using Trinity [24] (trinityrnaseq-r2013-02-25) with default values and kept all contiguous segments (contigs) with length > 100 bp. These contigs were then aligned using blastn (megablast with default values and an e-value of e-05) against the RefSeq viral database. Contigs with a viral hit were further aligned against the nt database and analyzed as in the two previous steps.

In pipeline 4 we used TriageTools [25] (v0.2.0), a tool that efficiently screens input reads for similarity to a specific target sequence. TriageTools was utilized with default parameters, the raw reads as input and as target the RefSeq sequences of HPV types 16 and 18 and HHV types 4 and 5. At a later stage we added to the pool of viruses, HHV 6B, Merkel cell polyomavirus and high-risk HPV viruses (as explained in the Results section). After obtaining the hit reads from TriageTools (reads that match the target sequence), we aligned them to the corresponding viral genome (target sequence) with BWA. All reads aligned to the viral genome where further aligned against the nt database with blastn and analyzed as in the previous steps.

In the pipeline 5, VirusSeq [19], a published bioinformatics pipeline for the detection of viruses was used. VirusSeq accepts the raw reads as input. Although it uses an empirical cut-off of 1000 reads for virus detection, the user can manually examine the number of reads mapped to each viral genome included in the database and identify the viral reads of his/her interest (even with a number of reads below this cutoff).

### Computational detection of viral sequences using Exome-Seq

We applied pipelines 1–4 to those samples where true viral sequences were detected in the RNA-seq reads. All pipelines (with the exception of pipeline 1) were adjusted to better fit the larger read size of the exome sequences. In the second pipeline we used megablast instead of blastn. In the third pipeline we performed a *de novo* assembly with Trinity on the unmapped reads as previously described, however we increased the length of the contigs to >200 bp. In the fourth pipeline we increased the *hits* parameter to 72 instead of the default 36.

### Positive control sequences

As it is essential to test the efficiency of the different pipelines, we decided to use a control dataset of RNA sequences as a positive control. We obtained the hepatocellular carcinoma RNA-Seq test set accompanying the VirusSeq algorithm. In the VirusSeq publication the same set was used for testing purposes and hepatitis B virus transcripts and viral integration loci were detected. The different pipelines were tested with this test set in order to identify the most abundant virus present.

### EBV IHC

For each sample, a representative FFPE block containing invasive adenocarcinoma was selected and a 4 μm-thick slice was cut.

EBV IHC was performed as follows: briefly, sections were de-paraffinized and processed using the Ventana detection system with the iViewTM DAB detection kit (Ventana, Tucson, Arizona). Antigen retrieval was performed with EDTA (Tris/borate/EDTA; pH 8.4). The slides were then incubated in a prediluted solution of Monoclonal Mouse Anti-EBV, LMP Clone CS.1-4 (DAKO, code IS753) at room temperature for 32 minutes. After staining, slides were processed in accordance with routine protocols.

### DNA extraction

DNA from FFPE material was extracted using QIAamp DNA FFPE Tissue kit® (Qiagen,) following the manufacturer’s instructions. DNA concentration was measured using the NanoDrop 1000 instrument (Thermo Scientific). All samples yielded enough material for downstream analyses.

### HPV PCR

PCR was performed using the Cobas® 4800 HPV Amplification/Detection kit (Roche, Basel, Switzerland) on the COBAS Z4800 instrument. This test is a qualitative in vitro test for the detection of HPV in the clinical setting. The test utilizes amplification of target DNA by PCR and nucleic acid hybridization for the detection of 14 high-risk (HR) HPV types in a single analysis. The test specifically identifies (types) HPV 16 and HPV 18 while concurrently detecting the rest of the high-risk types (31, 33, 35, 39, 45, 51, 52, 56, 58, 59, 66 and 68) at clinically relevant infection levels. ß-globin was used as an internal control.

## Results

### Five virus detection pipelines successfully recovered hepatitis B virus (HBV) transcripts in a hepatocellular carcinoma

**Figure 1:**
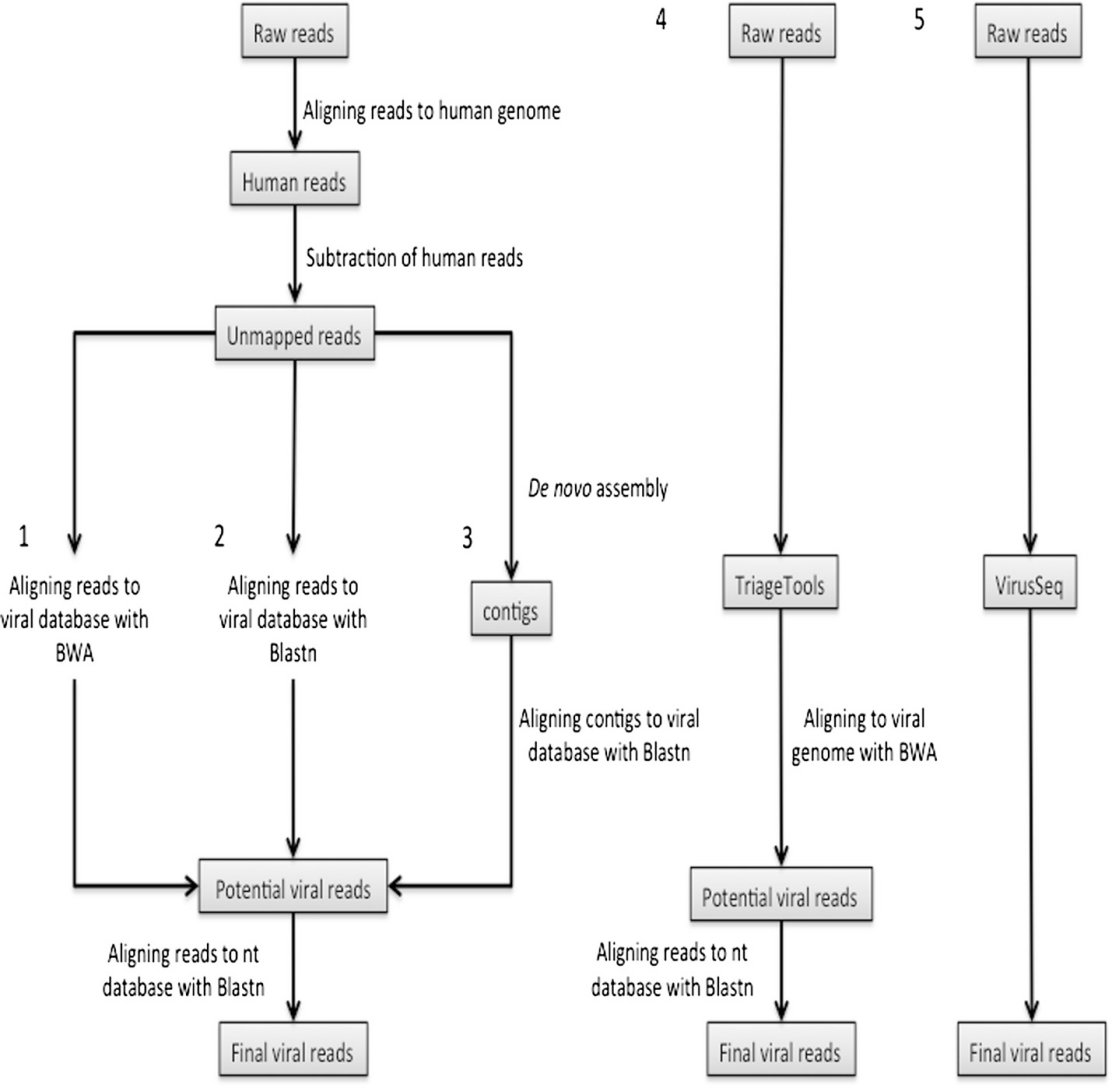
**Computational pipelines for the detection of non-human sequences**. Five different pipelines were used in this study. In pipelines 1, 2 and 3 raw reads were first aligned to the human genome and the unmapped reads were then used for alignment to the viral database with BWA, for alignment to the viral database with BLAST, and for *de novo* assembly and alignment to the viral database, respectively. In pipelines 4 and 5, raw reads were given as input to TriageTools and VirusSeq, respectively.

The four in-house and one published virus detection pipelines we implemented are depicted in Figure 1. Pipelines 1–3 and 5 first select reads that cannot be mapped to the human genome and attempt to map them on the RefSeq viral genome database and Gib-V database, respectively. In pipelines 1–3, reads with a viral hit are then screened against a comprehensive general-purpose sequence database (NCBI’s Non Redundant, NR) in order to further rule out their human origin. Reads with better BLAST hits to human than viral sequences are discarded. Pipelines 1–3 use the same initial alignment step and the same final filtering step (see Methods). However, they rely on different strategies for the viral sequences alignment. Pipeline 1 uses BWA, a standard short read aligner in NGS studies; pipeline 2 uses BLAST, a decade-old, proven aligner; pipeline 3 attempts to circumvent the limits of short reads by inserting a *de novo* assembly step before the viral screening. Pipeline 5 (VirusSeq) is conceptually similar to pipeline 1, but was set up by an independent team who took different technical routes at all steps of the implementation.

Pipelines 1–3 and 5 are unbiased with respect to which virus may be associated with breast, and potentially other, cancers. This valuable property comes at the cost of aligning the transcriptomes onto the human genome—an error-prone computational task. By contrast, pipeline 4 implements a targeted search limited to 4 viruses putatively associated with breast cancer, but it eliminates the initial human genome-mapping step. Instead, the transcriptome reads mapping to target viral genomes are selected directly with TriageTools, an *in silico* equivalent of the *in vitro* hybridization-based sequence capture.

As a positive control, we ran the five pipelines on a published RNA-Seq test set of hepatocellular carcinomas provided by the VirusSeq tool. All of the pipelines correctly detected HBV as the most abundant virus, representing 0.1‰ of all reads (~50,000 reads) with pipelines 1–2, 4 and 5. Pipeline 3 assembled two HBV contigs of 3129 bp and 170 bp.

### Limited evidence for viral transcripts for 3 in 58 breast cancer transcriptomes

Illumina HiSeq2000 transcriptome sequencing was performed on 58 breast cancers, producing a median of 64,792,160 2x50 bp reads per samples. The five pipelines described above were applied; the obtained results are provided in Table 1.

**Table 1:** **Viral sequences detected in the samples with the use of several computational methods**

Sample	Age	Raw Reads	Pipeline1 viral reads	Pipeline 2 viral reads	Pipeline 3 viral contigs	Pipeline 4 viral reads	Pipeline 5 viral reads	Virus Name	EBV IHC	HPV PCR
Test Set	NA	424484976	46821	50681	2	45071	43466	HBV		
HER2-13	46	64893130	0	0	0	0	0		negative	negative
HER2-14	49	50499646	0	0	0	0	0		negative	**positive high risk**
HER2-15	45	61599108	0	0	0	0	0		negative	negative
HER2-16	58	61491812	0	0	0	0	0		negative	negative
HER2-18	38	67873006	0	0	0	0	0		negative	negative
HER2-19	60	67894004	**22 (27)**	**25 (27)**	**0 (0)**	**17 (27)**	**26**	HHV6	negative	negative
HER2-2	83	59452650	0	0	0	0	0		negative	negative
HER2-20	66	85761124	0	0	0	0	0		negative	negative
HER2-21	46	93380848	0	0	0	0	0		negative	negative
HER2-22	39	50262908	0	0	0	0	0		negative	negative
HER2-23	55	68452272	0	0	0	0	0		failure	negative
HER2-24	49	45799400	0	0	0	0	0		negative	negative
HER2-3	35	59739682	0	0	0	0	0		negative	negative
LA-18	63	50984238	0	0	0	0	0		negative	negative
LA-19	37	83958354	0	0	0	0	0		negative	**positive hpv16**
LA-20	50	32869722	0	0	0	0	0		negative	negative
LA-21	51	76552678	0	0	0	0	0		negative	negative
LA-22	62	60308476	0	0	0	0	0		negative	negative
LA-23	73	42225858	0	0	0	0	0		negative	negative
LA-24	63	39585066	0	0	0	0	0		negative	NA
LA-25	58	49913768	0	0	0	0	0		negative	NA
LA-26	66	72421722	0	0	0	0	0		negative	negative
LA-27	62	66523668	0	0	0	0	0		negative	negative
LA-28	64	64969514	0	0	0	0	0		negative	NA
LA-29	51	23434748	0	0	0	0	0		negative	negative
LA-31	85	79158278	0	0	0	0	0		negative	NA
LA-32	58	68369496	0	0	0	0	0		negative	NA
LA-33	68	75436316	0	0	0	0	0		NA	negative
LA-4	61	64916390	0	0	0	0	0		negative	negative
LB-1	42	70818118	0	0	0	0	0		negative	negative
LB-3	53	44475406	0	0	0	0	0		negative	negative
LB-5	42	58896182	0	0	0	0	0		NA	NA
LB-15	79	37855250	0	0	0	0	0		negative	negative
LB-17	57	71008672	0	0	0	0	0		negative	negative
LB-18	61	56839562	0	0	0	0	0		negative	NA
LB-19	34	66451162	**2 (4)**	**2 (4)**	**0 (0)**	**2 (4)**	**2**	EBV	negative	negative
LB-20	45	114639766	0	0	0	0	0		negative	negative
LB-21	51	64647066	0	0	0	0	0		negative	negative
LB-22	53	79028228	0	0	0	0	0		negative	negative
LB-23	55	116825666	0	0	0	0	0		negative	negative
TN-1	41	75308960	**3 (0)**	**3 (0)**	**0 (0)**	**2(0)**	**3**	Merkel cell polyomavirus	negative	negative
TN-2	47	107171302	0	0	0	0	0		NA	NA
TN-3	59	34339412	0	0	0	0	0		negative	negative
TN-5	39	44460594	0	0	0	0	0		negative	negative
TN-15	35	56793532	0	0	0	0	0		NA	NA
TN-16	52	71331804	0	0	0	0	0		failure	negative
TN-17	34	81351348	0	0	0	0	0		negative	negative
TN-18	62	84104974	0	0	0	0	0		negative	negative
TN-19	50	19621240	0	0	0	0	0		negative	negative
TN-20	51	80751454	0	0	0	0	0		negative	negative
TN-21	56	65225662	0	0	0	0	0		negative	negative
TN-22	39	63983318	0	0	0	0	0		negative	negative
TN-23	65	50443546	0	0	0	0	0		negative	negative
TN-24	63	70061346	0	0	0	0	0		negative	negative
TN-25	79	43789446	0	0	0	0	0		negative	negative
TN-26	81	56344394	0	0	0	0	0		negative	negative
TN-27	57	64691190	0	0	0	0	0		negative	negative
TN-28	54	46437934	0	0	0	0	0		negative	negative

The numbers in the parenthesis indicate the number of sequences detected in exome-seq analysis. Viral hits are highlighted in bold.

In the first step of pipelines 1–3, the raw reads were mapped to the human genome with a median of 1,029,23 unmapped reads remaining for further viral sequences scanning. The most abundant non-human sequences detected by pipelines 1–3 were from Enterobacteria phage phiX174. This phage is used as a control during sequencing and should be viewed as a positive control, not a biologically relevant finding. A small number of reads were detected by pipelines 1, 2 and 5 that were mapped to human viruses by their full length and were considered as of true viral origin: 1) Two reads mapped to EBV in one sample; 2) three reads mapped to the Merkel cell polyomavirus in one sample; 3) a total of 22–26 reads mapped to human herpesvirus 6B (HHV 6B) in one sample. The *de novo* approach (pipeline 3) could not assemble viral sequences because of their small number.

In addition to the above findings, VirusSeq (pipeline 5) reported glyptapanteles flavicoxis bracovirus as the most abundant virus in all samples. This non-human false positive hit comes from the alignment of sequences with long stretches of “TG”.

The targeted approach (pipeline 4) was run initially with HPV 16 and 18 and EBV and CMV as targets. At a later stage we added to the list of viruses HHV 6B and Merkel cell polyomavirus as these two viruses were detected by the unbiased methods. High-risk human papillomaviruses were also added since DNA sequence was detected in one sample by PCR (see below). All hits from unbiased approaches, but not the PCR hits (see below), were recovered.

### Viral expression confirmed by exome sequencing but not confirmed by PCR or IHC

To validate these results, we ran the approaches 1–4 using exome sequences on the three samples where viral sequences were detected in the transcriptome sequencing data/reads. Pipelines 1, 2 and 4 successfully detected four reads of EBV and 27 reads of HHV 6B. Merkel cell polyomavirus was not recovered in the exome data by any of our approaches. As previously, the pipeline 3 could not assemble any viral sequences.

We next sought for the presence of EBV using IHC in 54 of the samples, and for the presence of HPV sequences using PCR in 49 samples. No EBV viral DNA was detected in any of the samples, including those positive with the transcriptome sequencing assay. On the contrary, we identified one positive sample for HPV 16, and one with high risk HPV strain, although no HPV sequence could be identified by both transcriptome and exome sequencing in these samples.

## Discussion

In order to explore the potential role of viral infection in breast cancers, we investigated their transcriptome sequences with five different *in silico* detection methods while in parallel we performed exome sequencing, IHC and PCR to detect viral infection at the DNA level.

The first three *in silico* methods (pipelines 1–3) have been so far exploited for the detection of viral sequences in human samples both alone or in combination. We also used a tool specifically designed for virus detection (VirusSeq, pipeline 5) and a bioinformatics tool designed for a more general purpose that could be applied in this study (TriageTools, pipeline 4). TriageTools differs from all the other methods since it searches for viral sequences in the raw sequenced reads without the need of first aligning them to the human reference genome, a step that it is known to be time-consuming. Using four of our alignment techniques, we detected viral sequences from EBV (2 reads), HHV6 (17–25 reads) and Merkel cell polyomavirus (2–3 reads). However, the number of detected viral sequences was orders of magnitude lower than in our positive control, an HBV-associated hepatocellular carcinoma transcriptome containing ~50,000 HBV reads. After normalizing by the sequencing coverage, viral reads represented at most 0.0004‰ of all the reads in our samples, compared to 0.1‰ in the positive control. Given the high prevalence of HHV 4 and 6, contamination, rather than a productive viral infection, is a possibility.

In parallel, we performed IHC on slides obtained from FFPE blocks from the same patients to detect the presence of EBV. In addition, the presence of HPV was investigated with a PCR assay routinely used in the clinic. No sample tested positive for EBV. Two samples were tested positive for HPV, although no HPV sequences were found in the transcriptomes and exomes of these samples. These discrepancies could result from a higher sensitivity of the PCR-based assay. It is also possible that the viruses identified with PCR are integrated in the DNA of these two patients, but not expressed, or it can be the result of the already reported heterogeneity of viral integration in human exome [26]. Heterogeneity in the distribution of the HHV 4 genome between regions of the same tumor as well as among different tumors was observed by Arbach et al. [27]. Additionally, they have found differences in the viral load, when they focused within one region or in the whole tumor. Although, a very low viral load is possibly not being detected by PCR, the advantages of next generation sequences would allow the detection of viral sequences even in small numbers thus making our techniques suitable for detecting small viral loads.

All our methods were successful at detecting viral sequences in the test set, but only a small number of viral sequences were detected in our breast cancer samples. A possible explanation could be a hit-and-run mechanism, where a virus infects the target tissue, performs a mutagenic action that makes the cell malignant and is then lost and therefore not detectable [28]. However, cases of hit-and-run mechanism are very difficult to detect. Another explanation is the fact that some viruses can contribute to carcinogenesis without being expressed, as is the case of the MMTV. In cases such as the two above, the RNA-seq technology is limited by the fact that it can only detect expressed viral sequences.

Although very few viral sequences of those known viruses were detected in our samples, further studies of other viruses could be of great interest. As mentioned in the recent review of Salmons et al. [29], there is accumulating evidence that retroviruses like MMTV are associated with breast cancer. However, retroviruses that are already integrated in the human genome (human endogenous retroviruses) could be of potential interest as well. Evidence of polymorphism in the integration of such endogenous viruses, as shown by Marchi et al. [30] and studies showing the detection of those sequences in breast cancer, could lead to a new viral agent linked to breast cancer.

## Conclusions

Our study demonstrates the ability to detect expressed viral sequences in breast cancer samples. The small number of those sequences indicates that there is no high enough expression to be able to conclude for a viral cause for breast carcinogenesis in our samples. However, it does not exclude the presence of integrated but silent viral sequences in the breast tumor genome or a possible hit-and-run mechanism. Further similar studies using whole genome sequencing are warranted on this subject.
